# MRPL3 is identified as a prognostic biomarker and therapeutic target in lung adenocarcinoma via a lactylation-disulfidptosis gene signature model and experimental validation

**DOI:** 10.3389/fimmu.2026.1772955

**Published:** 2026-05-19

**Authors:** Lan Yin, Rui Tang, Yue Song, Yunqiang Liu, Yongxin Ma

**Affiliations:** 1 Department of Medical Genetics, State Key Laboratory of Biotherapy, West China Hospital, Sichuan University, Chengdu, China; 2 Department of Medical Genetics, Frontiers Science Center for Disease-related Molecular Network, West China Hospital, Sichuan University, Chengdu, China

**Keywords:** biomarkers, disulfidptosis, lactylation, LUAD, Mrpl3, prediction of prognosis

## Abstract

**Background:**

Lung adenocarcinoma (LUAD), the most prevalent histological subtype of lung cancer, is a leading cause of global cancer mortality. Its pronounced heterogeneity poses a critical challenge, creating an urgent need for reliable biomarkers to accurately predict patient prognosis. Here, we focus on two critical tumor-promoting factors: lactylation and disulfidptosis.

**Methods:**

Differential expression analysis, correlation analysis, and univariate survival analysis were performed using gene expression profiles from The Cancer Genome Atlas (TCGA) and Gene Expression Omnibus (GEO) cohorts to screen differentially expressed genes (DEGs), lactylation and disulfidptosis related genes (LDRGs), and prognosis-related genes (PGs). A prognostic model was constructed via LASSO regression, with its efficacy evaluated using calibration plots and decision curve analysis (DCA).The regulatory role of mitochondrial ribosomal protein large subunit 3 (MRPL3) in lung adenocarcinoma (LUAD) progression was validated through both *in vivo* and *in vitro* experiments.

**Results:**

This model demonstrated reliable predictive performance across the testing set, validation set, and external validation cohorts. MRPL3 was found to be overexpressed in LUAD tissues and correlated with poor prognosis, advanced tumor stage, and an immunosuppressive tumor microenvironment. *In vitro*, MRPL3 knockdown inhibited LUAD cell proliferation and migration, reduced intracellular lactylation levels, and downregulated glycolysis-related proteins. Under glucose deprivation, MRPL3-knockdown cells exhibited a more stable F-actin cytoskeleton, suggesting evasion of disulfidptosis via XCT suppression. *In vivo*, MRPL3 knockdown significantly suppressed xenograft tumor growth.

**Conclusion:**

Collectively, this study establishes a robust prognostic model for LUAD and clarifies MRPL3’s role in regulating glycolytic-lactate metabolism and disulfidptosis to influence tumor progression and immune microenvironment remodeling. These findings provide a novel potential target and theoretical basis for LUAD prognosis assessment and targeted therapy.

## 1 Introduction

As the most common histological subtype of non-small cell lung cancer (NSCLC), LUAD ranks among the top contributors to cancer-associated deaths across the globe (1, 2). Despite advancements in early detection through low-dose computed tomography screening, a substantial proportion of patients are diagnosed at advanced stages with regional or distant metastases, contributing to the persistently poor prognosis associated with this malignancy (3). For those with advanced or metastatic LUAD, systemic therapies including targeted agents and immune checkpoint inhibitors have improved outcomes, yet durable responses are limited to a subset of patients, underscoring the need for novel therapeutic strategies and predictive biomarkers (4).

The metabolic reprogramming observed in LUAD, particularly the shift toward aerobic glycolysis, results in the accumulation of lactate in the TME, creating an acidic environment that can hinder effective immune responses and contribute to tumor progression (5, 6). Recent studies have revealed that lactate induces histone lactylation, a novel epigenetic modification that alters chromatin architecture and drives expression of genes involved in tumor proliferation, metastasis, and immune evasion (7, 8). In LUAD, elevated lactylation levels have been associated with malignant progression and poor survival (9).

Concurrently, disulfidptosis, a newly recognized form of regulated cell death triggered by disulfide stress and actin cytoskeleton collapse, has been implicated in LUAD pathogenesis (10, 11). This process is especially relevant under glucose-deficient conditions where NADPH shortage leads to abnormal disulfide bond accumulation in actin and other cytoskeletal proteins (10). Recent studies have shown that the high lactate flux driving lactylation depletes the cellular NADPH pool (12), creating a permissive environment for disulfidptosis. Furthermore, one study demonstrated that histone lactylation can promote the expression of the glutamate-cysteine ligase catalytic (GCLC) (13), and another study found that lactylation of the glutamate-cysteine ligase modifier (GCLM) enhances cellular antioxidant capacity (14). Therefore, a plausible hypothesis is that lactate-induced lactylation may serve as a dual epigenetic regulator in LUAD: on one hand promoting pro-tumorigenic gene expression programs, while on the other hand indirectly priming cells for disulfidptosis by altering redox balance and depleting NADPH. In general, targeting the lactylation-disulfidptosis axis may represent a novel therapeutic strategy for LUAD.

Given the potential cross-regulation between lactate-driven epigenetic changes and disulfide stress-induced cell death, we hypothesize that LDRGs may play a critical role in LUAD progression and therapy resistance. In this study, we aimed to establish a prognostic model based on LDRGs to predict clinical outcomes and immunotherapy response in LUAD patients. This integrative approach may provide new insights into the metabolic-epigenetic axis of cell death regulation and identify potential targets for LUAD treatment.

## 2 Materials and methods

### 2.1 Datasets

The RNA sequencing data and corresponding clinical information of LUAD were obtained from The Cancer Genome Atlas (TCGA) database (https://portal.gdc.cancer.gov/), including 513 primary tumor samples and 58 adjacent normal lung tissue samples. The microarray dataset GSE68465, which contains clinical and gene expression data from 443 patients with LUAD and 19 normal controls, was downloaded from the Gene Expression Omnibus (GEO, http://www.ncbi.nlm.nih.gov/geo/). Furthermore, A total of 1,544 lactylation-related genes (LRGs) (15, 16)and 887 disulfidptosis-related genes (DRGs) (10, 11, 17)were retrieved from previously published research (**Supplementary Table 1**).

### 2.2 Gene screening and functional annotation

Based on the gene expression profiles of TCGA-LUAD, we performed Pearson correlation analysis between 1,544 LRGs and 887 DRGs. Genes with an absolute correlation coefficient (|R|) > 0.5 and an adjusted p-value (padj) < 0.01 were identified as LDRGs. Differentially expressed genes (DEGs) between tumor and adjacent normal tissues were then identified using the “DESeq2” package, with the thresholds set as padj < 0.05 and |log2 fold change (FC)| > 1. The intersection of the LDRGs and the DEGs was further overlapped with the GEO expression dataset to obtain a set of consistently expressed candidate genes(CGs).

### 2.3 Prognostic gene identification

Univariate COX regression analysis was conducted separately in both the TCGA and GEO cohorts to identify prognostic genes from the CGs set (p < 0.05). The intersection of prognostically significant genes from both cohorts yielded the prognostic gene set (PGS) for downstream analyses. The intersections were visualized using the “circlize” package. Subsequently, tumor mutational burden (TMB) for each sample was calculated using the “Maftools” package. The protein–protein interaction (PPI) network of the prognostic gene was constructed using the STRING database (https://string-db.org/). Gene Ontology (GO) and Kyoto Encyclopedia of Genes and Genomes (KEGG) analyses were performed on the PGS using the “clusterProfiler” package.

### 2.4 Subtype identification and immune landscape characterization in LUAD

We applied the “ConsensusClusterPlus” package in R to classify the clinical subtypes of LUAD based on the PGS. Principal component analysis (PCA) was subsequently employed to evaluate the discriminative capacity of the prognostic gene set in distinguishing between the identified subtypes. Subsequently, we compared the differences between subtypes in terms of clinical variables (age, gender and stage) as well as overall survival (OS).

To further explore immune-related features, we compared tumor microenvironment (TME) scores and immune cell infiltration across subtypes. TME scores for all samples were calculated using the “estimate” R package, and immune cell infiltration was assessed using single-sample gene set enrichment analysis (ssGSEA) with the “GSVA” R package. Gene sets for immunosuppressive genes, immunostimulatory genes, and human leukocyte antigen (HLA) genes were retrieved from the TISIDB database (40), and their expression profiles were compared across subtypes (18).

### 2.5 Constructing risk model

We randomly divided the RNA sequencing expression profiles from TCGA into training and testing cohorts at a 1:1 ratio. In the training cohort, PGS were subsequently subjected to least absolute shrinkage and selection operator (LASSO) regression to refine the prognostic gene set. The retained genes, together with their corresponding regression coefficients, were used to construct a prognostic risk model. For each patient, a composite risk score was calculated as the sum of the expression levels of the selected genes weighted by their respective coefficients, and patients were stratified into high- and low-risk groups using the median risk score as the cutoff.

PCA, Kaplan-Meier (K-M) survival analysis and operating characteristic curve (ROC) analysis was performed to evaluate the performance of the model. Furthermore, associations between the risk score and clinical variables were examined to explore its clinical relevance. The robustness and generalizability of the risk model were validated in an independent external dataset (GSE68465).

### 2.6 Assessment of immunotherapeutic biomarkers

Somatic mutation data for LUAD were obtained from TCGA to calculate the TMB. Microsatellite instability (MSI) scores were downloaded via the “cBioPortalData” package. Samples with an MSI score above 0.4 were classified as MSI, while those below this threshold were considered microsatellite stable (MSS). The “easier” R package was used to generate immune response scores based on tumor microenvironment characteristics, with higher scores indicating greater potential sensitivity to immunotherapy (18).In addition, immunophenoscores (IPS) were downloaded from The Cancer Immunome Atlas (TCIA, https://tcia.at/home), and patients were stratified into two groups based on IPS values (≤8 vs. >8). Correlation analysis between the risk score and these biomarkers was performed to reflect the predictive value of risk scores on immunotherapy response.

### 2.7 Mendelian randomization analysis

We downloaded the full cis-eQTL summary statistics from the eQTLGen database (https://www.eqtlgen.org/cis-eqtls.html). Genome-wide association study (GWAS) summary statistics for LUAD (GCST004744) were obtained from the GWAS Catalog(https://www.ebi.ac.uk/gwas/), which included 11,273 LUAD patients and 55,483 control individuals of European ancestry. To construct the instrumental variables, we extracted eQTLs corresponding to the genes used in the risk prediction model and defined these as the exposure data. LUAD summary statistics were used as the outcome dataset. MR analyses were then conducted using the “TwoSampleMR” R package, which implements several MR approaches (e.g., inverse-variance weighted [IVW], MR-Egger, and weighted median methods) to estimate the causal effect of genetically predicted gene expression on LUAD risk.

### 2.8 Constructing a nomogram

Univariate and multivariate COX regression analyses were performed to identify independent prognostic factors associated with OS. A nomogram was constructed using the “survival” and “rms” packages. The predictive performance of the nomogram was evaluated using ROC curves and the corresponding area under the curve (AUC). Furthermore, decision curve analysis (DCA) was conducted to assess its potential clinical utility.

### 2.9 Drug sensitivity analysis

Drug sensitivity prediction was performed using a model trained on cell line data from the Genomics of Drug Sensitivity in Cancer (GDSC, https://www.cancerrxgene.org/). The oncoPredict package in R was applied to estimate drug sensitivity of LUAD patients in the TCGA cohort. A significance threshold of p < 0.05 was applied to identify drugs with statistically meaningful associations. For those drugs reaching significance, scatter plots were generated to visualize the correlation between MRPL3 expression levels and predicted drug sensitivity.

### 2.10 Analysis of core genes

Given the close link between lactate metabolism, lactylation, and mitochondrial function (18), we retrieved 1,136 mitochondrial genes (MTGs) from the MitoCarta 3.0 database (https://www.broadinstitute.org/). Core genes were identified by intersecting MTGs with those from our risk model. Their prognostic value and differential expression in LUAD were assessed using the KM-plotter database (https://kmplot.com/analysis/). Associations with tumor stage and immune subtypes were evaluated via TISIDB database (http://cis.hku.hk/TISIDB/), while relationships with the TME and immune cell infiltration were analyzed in R.

### 2.11 Cell culture and transfection

A549 and H1299 cell lines were obtained from Hycyte (Suzhou, China).Cells were cultured in RPMI 1640 medium supplemented with 10% fetal bovine serum (FBS) and 1% penicillin-streptomycin (Gibco, USA). An incubator containing 37 °C and 5% CO2 was used to grow cells. The knockdown and control plasmids of MRPL3 were purchased from MiaoLing Plasmid Platform (Wuhan, China). Transfection was performed using the jetPRIME reagent (Polyplus, France) following the manufacturer’s instructions.

### 2.12 Western blot and quantitative PCR

For western blotting, total protein extraction was performed using a universal protein extraction buffer (BioTeke, China) supplemented with a protease inhibitor mixture (Roche, Switzerland). Protein samples were separated using 7.5% SDS-PAGE and transferred onto a polyvinylidene fluoride (PVDF) membrane (Epizyme, China). The membrane was then blocked at room temperature with 5% non-fat milk for 1 hour. Subsequently, the membrane was incubated with the primary antibody, followed by incubation with a HRP-conjugated secondary antibody. Protein bands were detected using the Touch Imager chemiluminescence imaging system (e-BLOT, China). The primary antibodies used included anti-MRPL3 (Abmart), anti-XCT (Proteintech), anti-LDHA (HUABIO), anti-GLUT1 (HUABIO), and anti-Actin (HUABIO). Total RNA was extracted using the SteadyPure Quick RNA Extraction Kit (Accurate Biology, China). Subsequently, reverse transcription was performed with the HiFiScript gDNA Removal RT MasterMix (CWBIO, China). qPCR was carried out using the SuperStar Universal SYBR Master Mix (CWBIO, China) on a CFX Opus 96 real-time PCR system (Bio-Rad, USA). The primer sequences used were as follows: MRPL3-F: 5’-TACCGGGAACTTGGATTGCC-3’, MRPL3-R: 5’-GCATAAAGAGGAGTGCCTGGT-3’.

### 2.13 Cell proliferation, migration, and lactate assay

Cell viability under normal culture and glucose deprivation conditions was assessed using the Cell Counting Kit-8 (CCK-8; CWBIO, China). The cells were cultured in 12-well plates for the wound healing assay. A wound was introduced when the cells reached confluence. After wounding, the medium was replaced with serum-free medium, and images were captured at 0 hours and 72 hours under the same field of view. In the Transwell migration assay, a total of 8000 treated cells were suspended in serum-free medium and seeded into the upper chamber, while the lower chamber contained medium supplemented with 10% FBS. After 72 hours of incubation, the cells were stained and observed under a microscope (Olympus, Japan) to assess migration. The lactate concentration in cells was determined using the CheKineTM Micro Lactate Assay Kit (Abbkine, China). All procedures were performed in strict accordance with the manufacturer’s protocol.

### 2.14 Fluorescent staining of F−actin filaments

Cells were seeded onto sterile glass coverslips and cultured overnight to reach approximately 60–70% confluence. Glucose deprivation was then performed for 24 h. After treatment, the cells were washed twice with pre−warmed PBS, fixed with 4% paraformaldehyde for 15 min at room temperature, and permeabilized with 0.2% Triton X−100 for 5 min. The cells were subsequently incubated with phalloidin working solution (Phalloidin, Proteintech, China) for 20 min at room temperature to stain filamentous actin (F−actin). Nuclei were counterstained with DAPI (1µg/mL) for 5 min. After three washes with PBS, the coverslips were mounted onto glass slides using an anti−fade mounting medium. Fluorescence images were acquired using a confocal laser scanning microscope (Leica, Germany).

### 2.15 Animal experiments

All animal procedures were conducted in accordance with the Guide for the Care and Use of Laboratory Animals (National Institutes of Health Publication No. 80−23, revised 1996) and were approved by the Institutional Animal Care and Use Committee of West China Hospital, Sichuan University. 1×10^7^ MRPL3−knockdown or control H1299 cells were resuspended in 40μL of PBS, mixed with 40μL of Matrigel, and subcutaneously injected into 4−week−old female BALB/c nude mice. The mice were purchased from SPF Biotechnology Co., Ltd. (Beijing, China) and randomly divided into two groups (n = 5 per group). Tumor size and body weight were measured daily. Tumor volume (V) was calculated using the formula: V = length×width²/2. On day 28 post−inoculation, the mice were euthanized, and the xenograft tumors were excised and photographed.

### 2.16 Statistical analysis

Statistical analyses were performed using R (version 4.4.2) and GraphPad Prism (version 8.4.2). A p-value of less than 0.05 was considered statistically significant. Data are presented as the mean ± standard deviation (SD). Statistical comparisons between the two groups were performed using a two-tailed Student’s t-test. Survival rates were calculated by the Kaplan-Meier method, and differences between survival curves were assessed using the log-rank test.

## 3 Results

### 3.1 Prognostic gene signatures and functional insights

**Figure 1:**
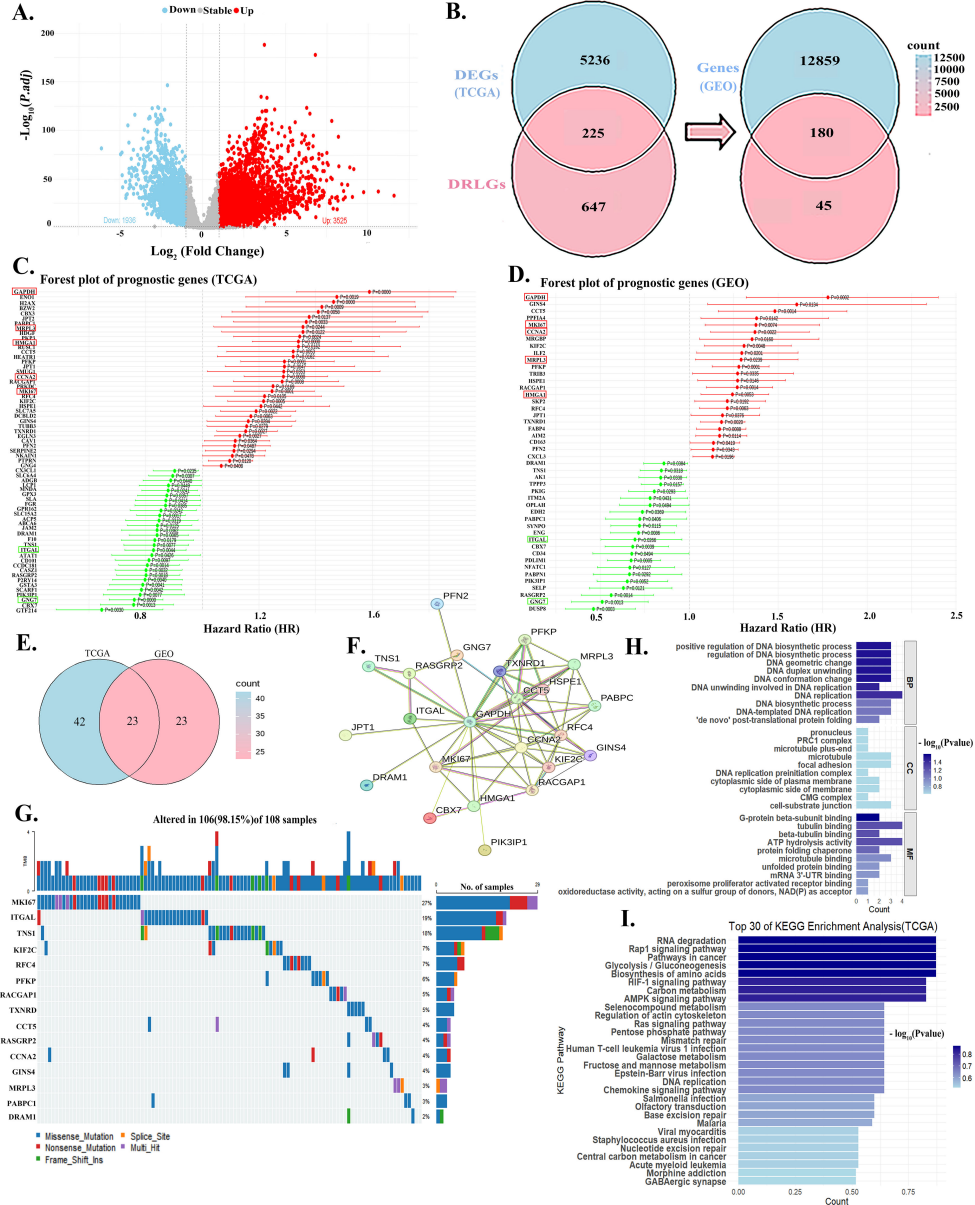
Identification and characterization of prognostic genes in LUAD. **(A)** The number of DEGs (n=5,461) identified in the TCGA-LUAD cohort. **(B)** Venn diagram illustrating the selection of CGs (n=180). **(C-E)** Univariate Cox regression analysis identified 23 PGs that were consistent in both the **(C)** TCGA and **(D)** GEO (GSE68465) cohorts. **(F)** PPI network of the 23 PGs, demonstrating close functional interconnections. **(G)** Mutation landscape of the PGs. **(H)** GO terms and **(I)** KEGG pathway analysis suggest the PGs are primarily involved in DNA synthesis and the Rap1 signaling pathway.

We identified 5,461 DEGs in the TCGA-LUAD cohort (**Figure 1A**). Correlation analysis identified 872 LDRGs, including 225 DEGs. After excluding 45 DEGs which were absent in the GSE68465 dataset, we retained 180 CGs. (**Figure 1B**). Univariate analysis consistently revealed 23 PGs in both TCGA and GEO cohorts (**Figures 1C–E**). PPI network demonstrated close interconnections among these genes (**Figure 1F**). Mutation analysis indicated that only 108 samples carried mutations of the PGs. The top 15 most frequently mutated genes accounted for 98.15% of these cases, with MKI67 exhibiting the highest mutation frequency (27%) (**Figure 1G**). GO and KEGG enrichment analyses further suggested that PGs are predominantly involved in DNA synthesis and may play an important role in the Rap1 signaling pathway (**Figures 1H, I**).

### 3.2 Clinical features of the two LUAD subtypes

**Figure 2:**
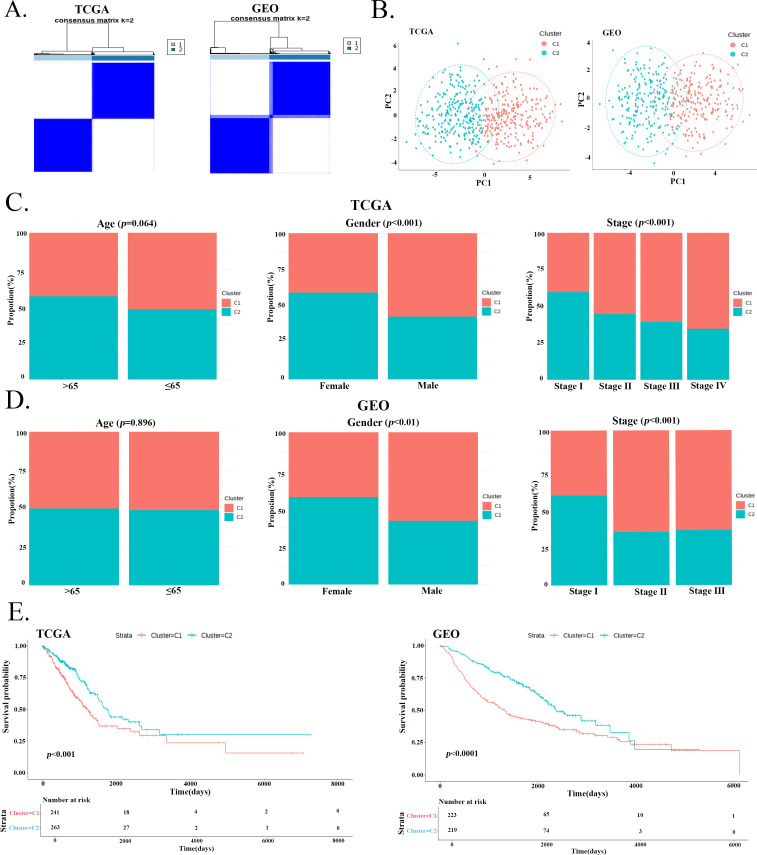
Clinical characterization of two LUAD subtypes identified by PGs expression. **(A)** Identification of two subtypes (C1 and C2) by consensus clustering based on 23 prognostic genes. **(B)** PCA further confirmed the clear separation between the C1 and C2 subtypes. **(C, D)** Distribution of clinical features (age, gender, and clinical stage) between the C1 and C2 subtypes in the TCGA and GEO cohorts. **(E)** K-M survival curves comparing the OS of patients between the C1 and C2 subtypes in both the TCGA and GEO cohorts. Patients in the C1 subtype had a significantly worse prognosis.

Our analysis demonstrated that the expression patterns of the 23 PGs effectively stratified LUAD patients into two distinct subtypes (**Figure 2A**), and the PCA further confirmed this (**Figure 2B**). Analysis of clinical characteristics showed that age did not differ significantly between subtypes in either the TCGA-LUAD or GSE68465 cohort. However, significant differences were observed in gender and clinical stage distribution. Specifically, the C2 subtype was enriched for female patients and early-stage (Stage I & II) cases (**Figures 2C, D**). K-M survival analysis demonstrated that patients in C1 group showed significantly worse prognosis in both cohorts (**Figure 2E**).

### 3.3 Two subtypes of the immune landscape

**Figure 3:**
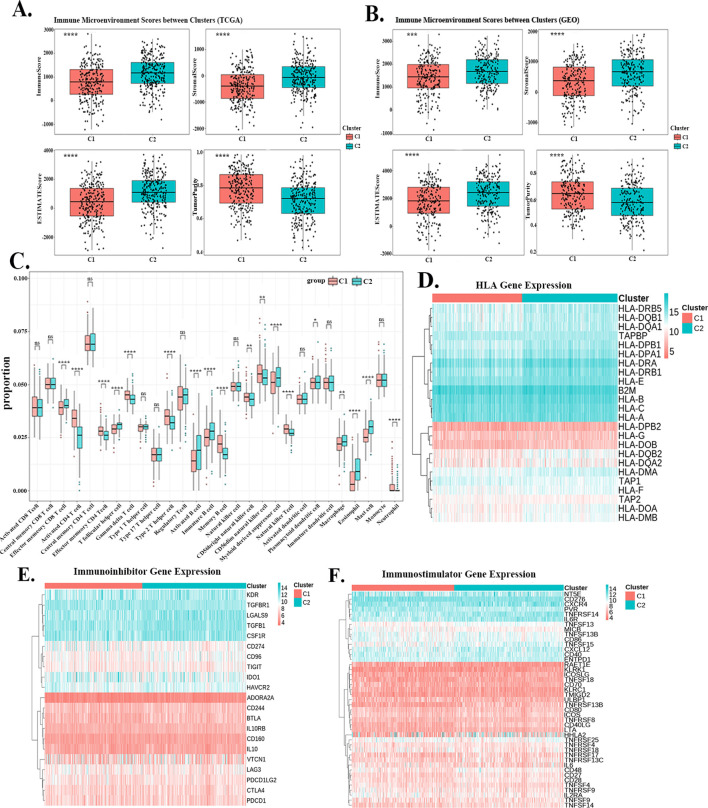
Immune landscape of the two LUAD subtypes. **(A, B)** Comparison of TME. scores between the C1 and C2 subtypes in the **(A)** TCGA and **(B)** GSE68465 cohorts. **(C)** Differential immune cell infiltration patterns between the two subtypes. **(D–F)** Heat map of the difference in expression levels of HLA genes, immunoinhibitory, and immunostimulatory in the two subtypes. “*” p<0.05, “**” p<0.01, “***” p<0.001, “****” p<0.0001.

In both the TCGA and GSE68465 cohorts, the C2 group exhibited significantly higher scores for all TME components except for tumor purity (**Figures 3A, B**). The immune cell infiltration patterns also differed significantly between the two subtypes (**Figure 3C**). In the C1 group, the levels of Effector memory CD8+ T cell, T follicular helper cell, Activated B cell, Immature B cell, Myeloid-derived suppressor cell (MDSC), Plasmacytoid dendritic cell, Macrophage, Eosinophil, and Mast cell were lower compared to the C2 group. Conversely, Neutrophil, Natural killer T cell, CD56dim natural killer cell, CD56bright natural killer cell, Memory B cell, Type 2 T helper cell, Gamma delta T cell, Effector memory CD4+ T cell and activated CD4+ T cells were significantly higher in the C1 group. The heatmaps (**Figures 3D–F**) clearly illustrated that the expression levels of most HLA genes, immunoinhibitory genes, and immunostimulatory genes were lower in the C1 group.

### 3.4 Constructing risk model

**Figure 4:**
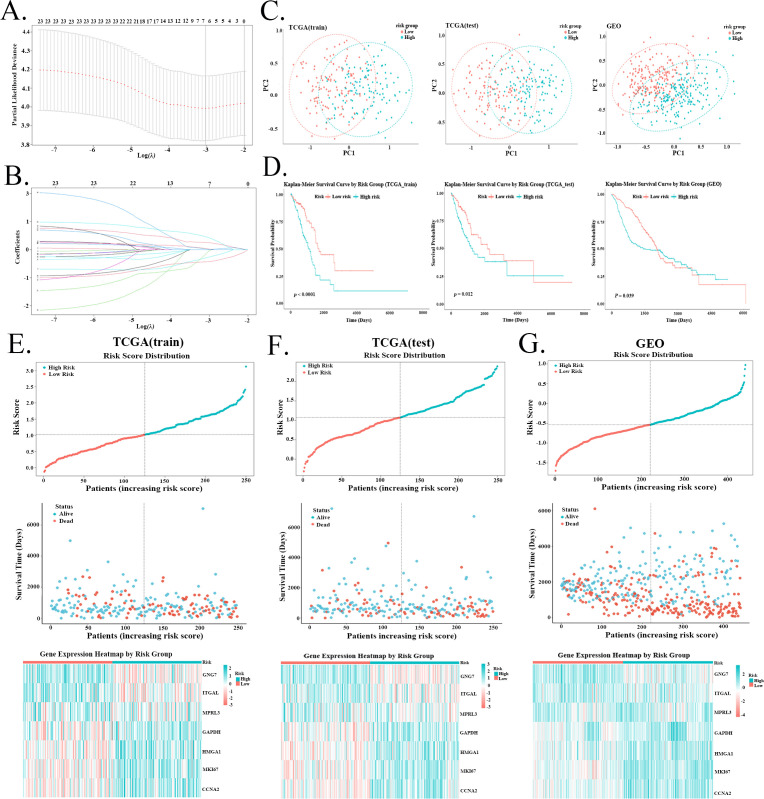
Risk model based on 11 genes. **(A, B)** LASSO regression identified seven RGs in the training set to construct the risk model. **(C)** PCA demonstrates a clear separation between the high-risk and low-risk groups based on the expression of the seven RGs. **(D)** K-M survival analysis confirms that patients in the high-risk group have significantly shorter OS than those in the low-risk group. **(E–G)** Distribution of survival status, survival time, and model gene expression levels in relation to the risk score for the TCGA training set, TCGA testing set, and GSE68465 cohort.

In the risk model, LASSO regression analysis identified seven RGs in the training set (**Figures 4A, B**). Univariate analysis showed that except for GNG7 and ITGAL, the other five RGs were significantly associated with poorer prognosis in both the TCGA and GSE68465 cohorts (**Figures 1C, D**). PCA revealed a clear separation between the high-risk and low-risk groups based on the expression of these seven RGs (**Figure 4C**). K-M survival curves confirmed that patients in the high-risk group had significantly shorter OS than those in the low-risk group (**Figure 4D**). Furthermore, **Figures 4E, F, and G** showed how the survival status and the expression of the RGs each associate with the risk score, a pattern that was consistent in all cohorts.

### 3.5 Prognostic risk characteristics and prediction of immunotherapy response in LUAD

**Figure 5:**
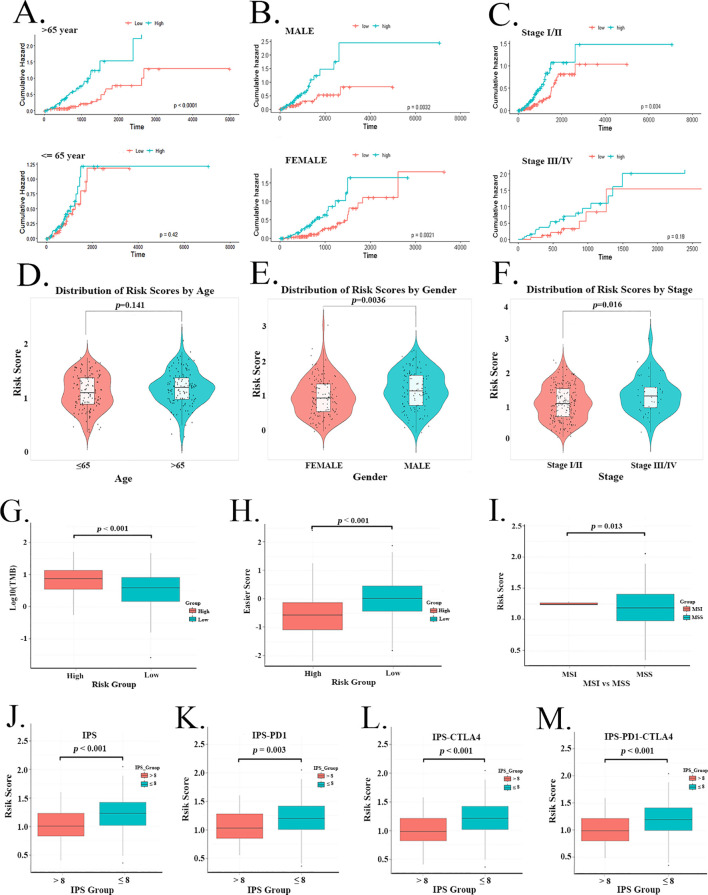
Prognostic risk characteristics and prediction of immunotherapy response in LUAD. **(A–C)** Cumulative hazard over time for <=65 and >65 years of age, male and female, and Stage I/II and III/IV patients in the TCGA training set. **(D–F)** Distribution of risk scores according to **(D)** age, **(E)** gender, and **(F)** clinical stage. **(G)** TMB levels and **(H)** easier scores in high and low-risk groups. **(I)** Differences in risk scores between MSI and MSS groups. **(J–M)**The relationship between IPS grouping and risk scores.

The risk characteristics of different clinical variables in the TCGA training set (**Figures 5A–C**) were analyzed using Kaplan-Meier survival analysis with the log-rank test. In subgroups based on age (≤65 vs. >65 years), gender (male vs. female), and stage (stage I/II vs. stage III/IV), the high-risk group consistently exhibited an increasing cumulative risk over time and maintained higher risk levels compared to the low-risk group. However, this difference was not statistically significant in the subgroup of patients aged ≤65 years or in those with stage III/IV disease. We also observed variations in risk scores within each clinical variable. Although the difference between age groups was not statistically significant, male patients and those with advanced disease (stage III/IV) tended to have higher risk scores (**Figures 5D–F**). Analyses of risk characteristics based on clinical variables in the TCGA testing set and the GSE68465 cohort were provided in **Supplementary Figure 1**.

Patients with LUAD were analyzed based on TMB, MSI, IPS, and easier scores to predict their response to immunotherapy. The high-risk group was characterized by significantly higher TMB and lower easier scores (**Figures 5G, H**). MSI was also associated with a higher risk score (**Figure 5I**). Furthermore, a strong inverse correlation was observed between the IPS and the risk score (**Figures 5J–M**).

### 3.6 Causal relationship and discriminatory power of RGs in LUAD

To explore the potential causal relationship between the seven RGs and LUAD, we performed MR analysis. The results indicated that only ITGAL and MKI67 exhibited a causal effect on LUAD susceptibility, with elevated expression of both genes associated with an increased risk of developing LUAD (**Supplementary Figure 2A**).The scatter plot and leave-one-out results are shown in **Supplementary Figure 3**, and the sensitivity analysis results are presented in **Supplementary Table 2**.

To further validate the prognostic model, we conducted PCA to assess the discriminatory ability of four different gene sets: the full gene set, CGs, PGs, and the seven RGs. In the TCGA training set, when considering all genes, the first three principal components (PCs) explained 11.05%, 7.84%, and 5.68% of the variance (cumulative 24.57%). In contrast, the CGs explained 20.83%, 9.42%, and 7.22% of the variance (cumulative 37.47%). The PGs accounted for 46.34%, 10.37%, and 5.62%, contributing to a total of 62.33% of the variance. Notably, PCA based on the RGs showed a markedly strong discriminatory power, with the first three PCs explaining 56.45%, 16.2%, and 8.69% of the variance, resulting in a cumulative explained variance of 81.34% (**Supplementary Figures 2B–E**). Consistent results were observed in the TCGA testing set (**Supplementary Figures 2F–I**)and the GSE68465 cohort(**Supplementary Figures 2J–M**), confirming the robust discriminative capacity of the RGs.

### 3.7 Prognostic model validation and nomogram construction in LUAD

**Figure 6:**
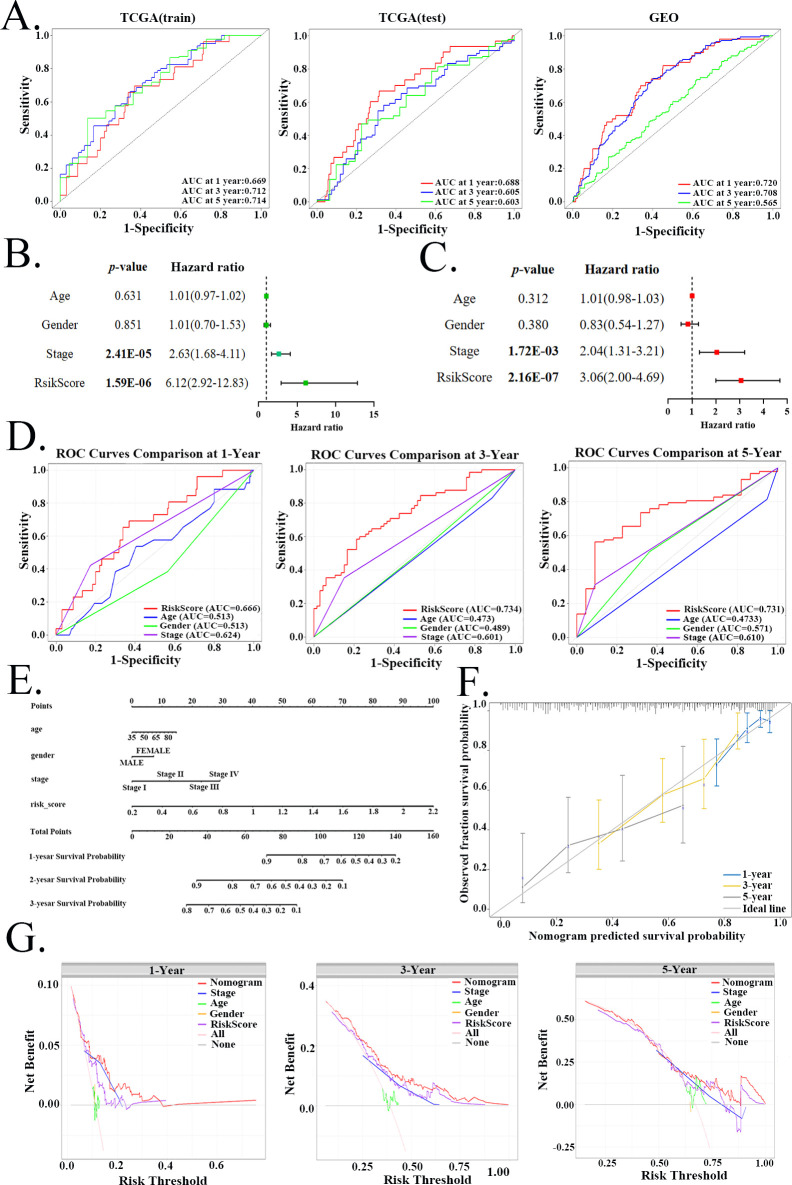
Prognostic model validation and nomogram construction in LUAD. **(A)** ROC curves assessing the predictive performance of the risk model for 1-, 3-, and 5-year overall survival across the TCGA training set, TCGA testing set, and the GSE68465 validation cohort. Forest plots from **(B)** univariate and **(C)** multivariate Cox regression analyses evaluating the risk score as an independent prognostic factor. **(D)** ROC curves comparing the predictive accuracy of the risk score against clinical variables (age, gender, stage) for 1-, 3-, and 5-year survival. **(E)** Nomogram constructed based on the TCGA training set. **(F)** Nomogram calibration plots for 1, 3, and 5 years. **(G)** DCA evaluating the clinical net benefit of the nomogram against individual clinical variables across different time points.

The ROC curves from three datasets (TCGA training set, TCGA testing set, and GSE68465) assess the performance of the prognostic model at 1-, 3-, and 5-year survival (**Figure 6A**). In the TCGA training set, the AUC values were 0.669, 0.712, and 0.714 for 1-, 3-, and 5-year survival, respectively. Corresponding AUC values in the TCGA testing set were 0.688, 0.605, and 0.603. In the external GSE68465 validation cohort, the model achieved AUCs of 0.720 and 0.708 for 1- and 3-year predictions, while the 5-year AUC was 0.565. Univariate Cox regression analysis revealed that the hazard ratio (HR) of the risk model was 6.12 (95% confidence interval(CI): 2.92-12.83, p<0.001), suggesting that the model may serve as an independent prognostic factor (**Figure 6B**). This result was further validated by multivariate analysis, which confirmed the model’s independent predictive value for clinical outcomes in LUAD patients (HR: 3.06, 95% CI: 2.00-4.69, p<0.001) (**Figure 6C**). ROC curve comparisons for 1-year, 3-year, and 5-year survival rates indicated that the AUC values for the risk score were 0.666, 0.734, and 0.731, respectively, surpassing those of age, gender, and clinical stage (**Figure 6D**).To develop a more practical personalized prognostic tool, a nomogram integrating the risk score, age, gender and clinical stage was constructed (**Figure 6E**). The calibration plot showed that the predicted probabilities closely matched the actual probabilities (**Figure 6F**). DCA demonstrated that the nomogram outperformed other clinical variables in predicting clinical outcomes at 1-year, 3-year, and 5-year time points (**Figure 6G**).Furthermore, validation with the TCGA testing set and GSE68465 dataset supported the model’s effectiveness as an independent prognostic factor (**Supplementary Figures 4**, **S5**).

### 3.8 Drug sensitivity analysis

**Figure 7:**
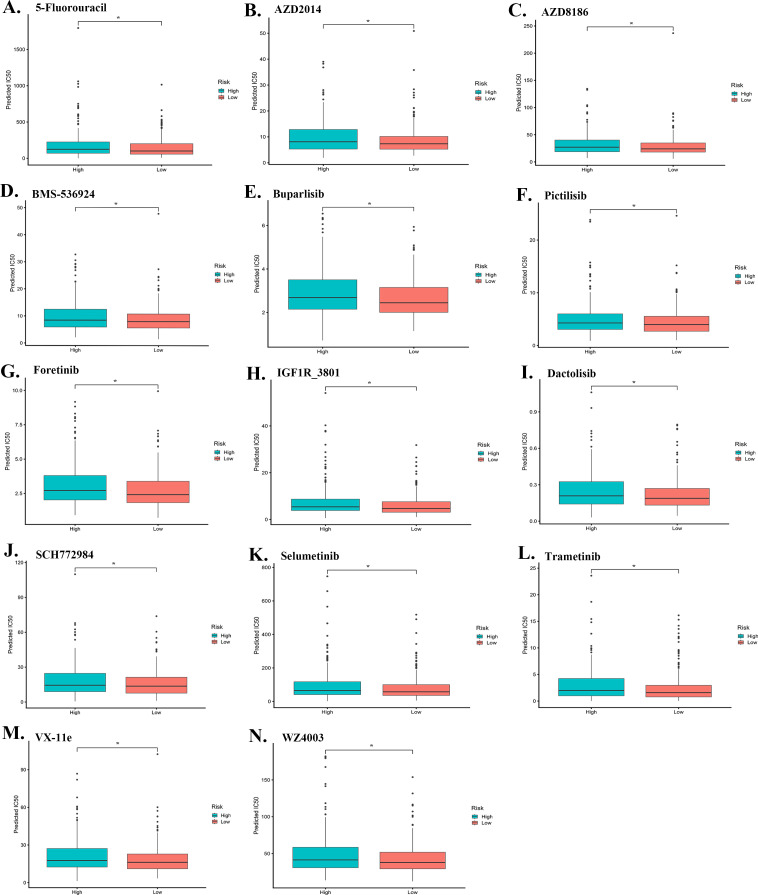
Drug sensitivity analysis. Drug sensitivity analysis demonstrated that the high-risk group exhibited significantly higher IC50 values for multiple drugs, including 5-Fluorouracil, AZD2014, AZD8186, BMS-536924, Buparlisib, Pictilisib, Foretinib, IGF1R-3801, Dactolisib, SCH772984, Selumetinib, Trametinib, VX-11e, and WZ4003 (P<0.05), indicating reduced drug sensitivity. **(A–N)** show the IC50 values for these drugs across the high-risk and low-risk groups.

The drug sensitivity analysis revealed that the high-risk group exhibited significantly increased IC50 values for 5-Fluorouracil, AZD2014, AZD8186, BMS-536924, Buparlisib, Pictilisib, Foretinib, IGF1R-3801, Dactolisib, SCH772984, Selumetinib, Trametinib, VX-11e, and WZ4003 (P<0.05), indicating reduced drug sensitivity (**Figures 7A–N**).

### 3.9 The role of the key gene MRPL3 in LUAD

**Figure 8:**
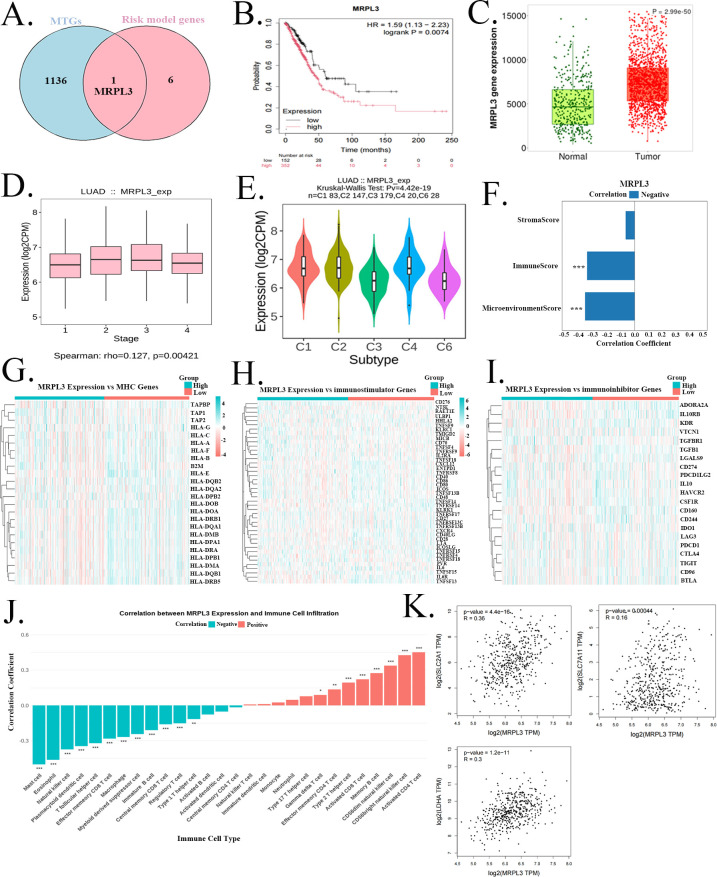
The role of the key gene MRPL3 in LUAD. **(A)** Identification of MRPL3 as a key risk gene among the seven-model signature. MRPL3 expression is significantly upregulated in LUAD tumors **(B)**, and high expression predicts poorer overall survival **(C)**. **(D)** MRPL3 expression levels in samples of different stages. MRPL3 expression stratified LUAD samples into five distinct immune subtypes **(E)** and showed a negative correlation with TME score **(F)**. Heatmaps displaying the expression patterns of **(G)** HLA genes, **(H)** immunostimulatory genes, and **(I)** immunoinhibitory genes in groups with high and low MRPL3 expression. **(J)** Correlation Between MRPL3 and Immune Cells. **(K)** Correlation of MRPL3 expression with GLUT1/SLC2A1, LDHA, and XCT/SLC7A11 expression.

Among the seven RGs, we identified MRPL3 (Mitochondrial Ribosomal Protein Large subunit 3), a key executor of mitochondrial function that may play a role in the development of LUAD (**Figure 8A**). MRPL3 expression was significantly upregulated in LUAD tumor tissues compared to normal counterparts, and elevated expression was associated with poorer clinical outcomes (**Figures 8B, C**). High MRPL3 levels were also correlated with advanced tumor stage (**Figure 8D**). To further characterize the clinical relevance of MRPL3 expression in LUAD, we analyzed its association with key clinicopathological features using the TCGA-LUAD dataset (**Supplementary Table 3**). MRPL3 expression was significantly positively correlated with pathologic stage (AJCC, FDR = 0.0029), pathologic T stage (FDR < 0.001), pathologic M stage (FDR = 0.0103), and pathologic N stage (FDR = 0.0151), indicating that higher MRPL3 expression is associated with more advanced tumor progression. In addition, elevated MRPL3 expression was significantly correlated with vital status (death, FDR = 0.029) and male gender (FDR = 0.0433), while no significant correlation was observed with age at diagnosis (FDR = 0.8227).

Further analysis revealed a close association between MRPL3 and the immune microenvironment. MRPL3 expression stratified LUAD samples into five distinct immune subtypes and showed a negative correlation with TME score (**Figures 8E, F**). Moreover, the high-MRPL3 group exhibited downregulation of most HLA genes, immunostimulatory genes, and immunoinhibitory genes (**Figures 8G–I**). As shown in **Figure 8J**, MRPL3 was negatively correlated with various immune cells, such as mast cells, eosinophils, and natural killer cells. Conversely, it was positively correlated with several immune cells, including activated CD4+ T cells, memory B cells, and activated CD8+ T cells. MRPL3 expression was positively correlated with the IC50 of drugs that exhibited differential sensitivity between the high- and low-risk groups (**Supplementary Figure 6**). Notably, MRPL3 showed statistically significant correlations with the IC50 of BMS-536924, Buparlisib, Foretinib, and Selumetinib (P<0.05).

### 3.10 MRPL3 depletion suppresses LUAD progression

**Figure 9:**
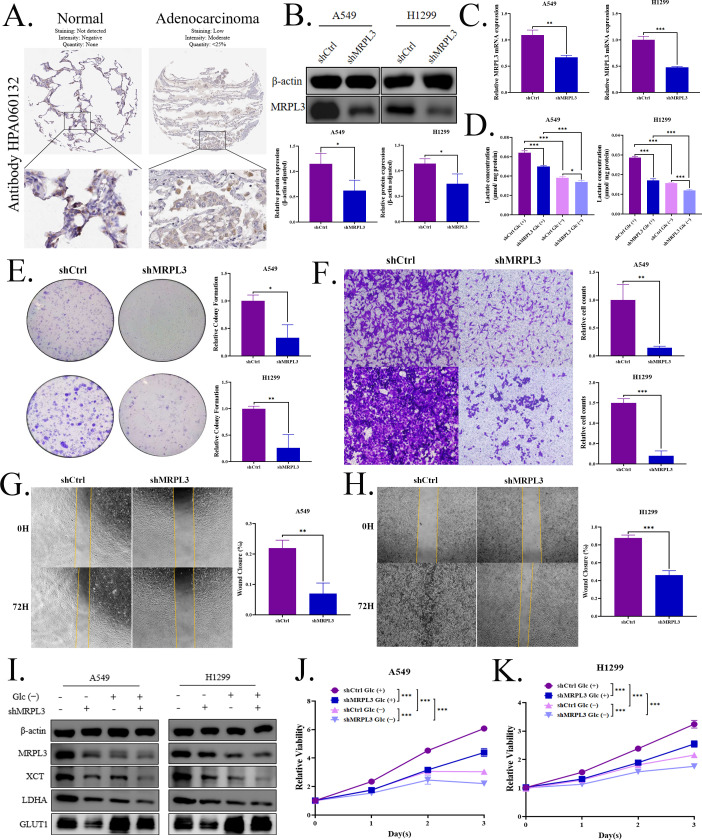
Experimental verification of MRPL3’s role in LUAD cells. **(A)** Immunohistochemical results showing elevated MRPL3 levels in LUAD tissues compared to normal tissues. **(B, C)** WB and qPCR analyses confirming successful knockdown of MRPL3 at both protein and mRNA levels in A549 and H1299 cells. **(D)** Reduced lactylation levels in MRPL3 knockdown cells. Colony formation **(E)**, transwell assays **(F)**, wound healing**(G, H)**, and CCK-8 assays **(J, K)** demonstrating inhibited cell proliferation and migration following MRPL3 knockdown. **(I)** Downregulation of GLUT1/SLC2A1, LDHA, and XCT/SLC7A11 proteins after MRPL3 knockdown. Effects of glucose deprivation on protein levels **(I)** of GLUT1, LDHA, MRPL3, and XCT, as well as lactate production **(D)** and cell proliferation **(J, K)**.

**Figure 10:**
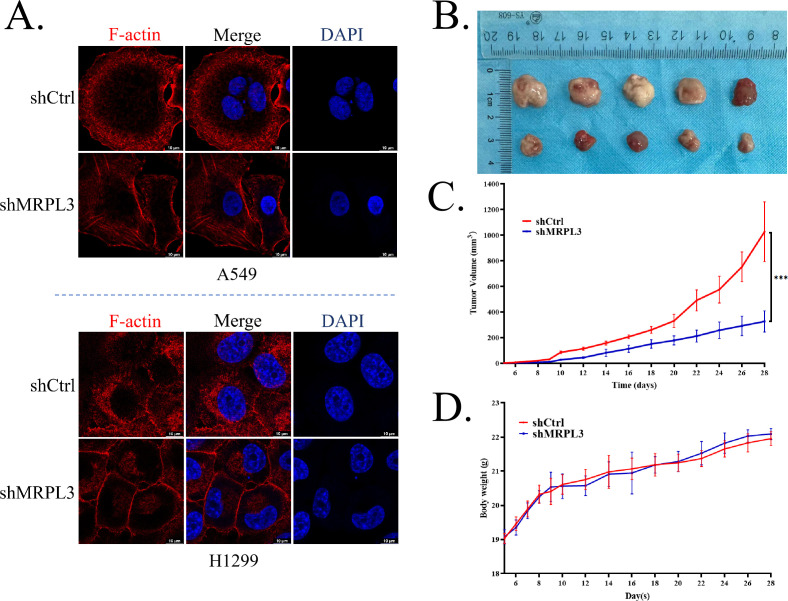
MRPL3 knockdown stabilizes F-actin and suppresses tumor growth. **(A)** Representative confocal images of F-actin (phalloidin staining, red) and nuclei (DAPI, blue) in cells cultured under glucose deprivation conditions for 24 h. Knockdown of MRPL3 reduces disulfidptosis sensitivity and results in a more stable F-actin. **(B)** Xenograft tumors in each group were shown. **(C)** Tumor growth curves of xenografts derived from control or MRPL3−knockdown H1299 cells subcutaneously injected into nude mice (n = 5 per group). Tumor volumes were measured daily. **(D)** Body weight curves of mice in the two groups during the experimental period. *P < 0.05, **P < 0.01, ***P < 0.001, according to Student’s t-test.

Immunohistochemical results from the HPA database further indicated elevated levels of MRPL3 in LUAD tissues compared to normal tissues (**Figure 9A**). WB and qPCR analyses confirmed the successful reduction of both MRPL3 protein and mRNA levels in the two knockdown models constructed in A549 and H1299 cells (**Figures 9B, C**). Knockdown of MRPL3 led to a decrease in lactylation levels in the cells (**Figure 9D**). Colony formation and CCK-8 assays demonstrated that MRPL3 knockdown significantly inhibited cell proliferation (**Figures 9E, J, K**). Furthermore, wound healing and transwell assays showed that cell migration was significantly suppressed after MRPL3 knockdown (**Figures 9F–H**). As shown in **Figure 9I**, protein levels of GLUT1/SLC2A1 and LDHA, which were closely associated with glycolysis and lactate production (19, 20), as well as the disulfidptosis-related gene XCT/SLC7A11 (21), were downregulated after MRPL3 knockdown. This was consistent with the correlation analysis results from the Gene Expression Profiling Interactive Analysis (GEPIA) database (http://gepia.cancer-pku.cn/index.html) shown in **Figure 8K**. Furthermore, under glucose deprivation conditions, we observed an upregulation of GLUT1 but a downregulation of LDHA, MRPL3, and XCT, accompanied by concomitant decreases in lactate levels and cell proliferation capacity (**Figures 9I–K**). Notably, under glucose starvation conditions, MRPL3-knockdown cells exhibited a more stable F-actin architecture (**Figure 10A**). To validate the effect of MRPL3 on LUAD growth *in vivo*, MRPL3-knockdown H1299 cells and control cells were further injected into nude mice. Tumors in the MRPL3-knockdown group grew slowly than the tumors of control group (**Figure 10C**), with no significant difference in body weight between the two groups (**Figure 10D**). The tumor volume in the MRPL3-knockdown group was significantly lower than that in the control group (**Figure 10B**).

## 4 Discussion

The remarkable heterogeneity of LUAD contributes to the considerable variation in clinical outcomes and therapeutic responses, highlighting the critical need for novel biomarkers to accurately predict prognosis (22). Recently, targeting metabolic reprogramming and regulating cell death modalities have emerged as promising strategies in cancer treatment (23, 24). Disulfidptosis, a newly discovered form of regulated cell death triggered by disulfide stress and cystine accumulation, offers a novel perspective for cancer therapy (10, 21). Liu et al. demonstrated that under glucose starvation conditions, cancer cells with high SLC7A11 expression undergo pronounced disulfide-induced actin cytoskeleton aggregation and cell death, revealing considerable potential for targeting cellular metabolism to induce specific modes of cell death (10). Meanwhile, the emerging field of lactylation bridges cancer metabolic reprogramming and the epigenetic landscape. Zhang et al. demonstrated that lactate induces lactylation of both histone and non-histone proteins, directly modulating gene transcription and playing a critical role in shaping the TME (16, 25). While both disulfidptosis and lactylation are increasingly implicated in tumor progression, their potential crosstalk and combined prognostic value in LUAD remain largely unexplored.

In this study, we report a robust prognostic model composed of seven LDRGs through the integration of multi-omics data and bioinformatics analysis. This model effectively predicts clinical outcomes, immune microenvironment characteristics, and treatment responses in LUAD. Thus, our findings provide a valuable prognostic tool and reveal potential biological mechanisms driving LUAD progression.

To begin our investigation, we identified 23 PGS consistently associated with prognosis across multiple cohorts through screening of TCGA and GEO datasets. These genes were significantly enriched in processes related to DNA synthesis and the Rap1 signaling pathway. The Rap1 pathway plays a crucial role in cell adhesion, migration, and proliferation, with its aberrant activation closely linked to the progression and metastasis of various cancers (26, 27). This suggests that the initial gene set captures the core biological processes underlying the malignant progression of LUAD.

Based on these 23 PGs, we successfully stratified LUAD patients into two subtypes with distinct clinical and immunological differences. The C1 subtype, associated with poorer prognosis, was enriched in male patients and those at advanced stages. This subtype exhibited a unique “immune desert” phenotype, characterized by overall reduced immune cell infiltration, impaired antigen presentation machinery, and a global downregulation of both immunostimulatory and immunosuppressive molecules (28). This immunosuppressive TME likely constitutes a fundamental reason for the unfavorable outcomes in C1 patients. Notably, although certain T-cell subsets were numerically increased in the C1 subtype, the concomitant low HLA expression and low immune scores suggest a state of T-cell dysfunction or exhaustion, rendering them incapable of effective tumor antigen recognition (29–31).

The risk model constructed using seven RGs is the central contribution of this study. This model demonstrated robust prognostic predictive ability across the training set, internal test set, and external validation cohort. Multivariate Cox regression analysis confirmed that the risk score is an independent prognostic factor, regardless of age, gender, and clinical stage. Moreover, its area under the ROC curve for predicting 1-, 3-, and 5-year survival rates outperformed traditional clinical indicators, highlighting its exceptional discriminative power. Additionally, the nomogram integrating the risk score, age, sex, and clinical stage demonstrated superior predictive performance for clinical outcomes at the 1-, 3-, and 5-year time points compared to other clinical variables. MR analysis further supported the biological foundation of the model by confirming that two risk genes, ITGAL and MKI67, were potentially causally related to the development of LUAD.

From a translational medicine perspective, this risk model demonstrated considerable potential for clinical application. Firstly, in predicting immunotherapy response, the high-risk group exhibited higher TMB, and groups with higher MSI were associated with elevated risk scores. Both TMB and MSI were commonly considered positive predictive indicators of immune checkpoint inhibitor efficacy (32, 33). However, the high-risk group paradoxically had lower easier scores and immune checkpoint IPS scores. This “high TMB but low immune infiltration” phenotype may be associated with immune evasion mechanisms and an immunosuppressive microenvironment within the tumor, which could suggest poor responses to current immunotherapies. This finding provides new insights into the understanding of primary resistance to immunotherapy (34–36).Secondly, drug sensitivity analysis suggested that high-risk patients may exhibit reduced sensitivity to various targeted therapies and chemotherapies, which provides valuable guidance for personalized treatment strategies in clinical practice.

We have extensively investigated the function of the key risk gene MRPL3. As a mitochondrial ribosomal protein, the high expression of MRPL3 is closely associated with poor prognosis, advanced tumors, and an immune-suppressive microenvironment (37). Its expression is negatively correlated with TME scores and is associated with widespread downregulation of various immune-related genes, suggesting that MRPL3 may indirectly remodel the immune microenvironment by regulating the metabolic state of tumor cells, thereby promoting tumor immune evasion. Furthermore, the positive correlation between MRPL3 expression and the IC50 values of multiple drugs further establishes its role as a potential therapeutic target and a biomarker for drug resistance. Knockdown of MRPL3 *in vitro* significantly inhibited the proliferation and migration of LUAD cells, confirming its functional necessity in the aggressive cancer phenotype. Furthermore, *in vivo* nude mouse xenograft assays demonstrated that MRPL3 knockdown markedly suppressed tumor growth, further supporting the above conclusion. Interestingly, MRPL3 knockdown not only resulted in an overall decrease in lactylation levels but also led to the downregulation of GLUT1 and LDHA protein levels, suggesting that MRPL3 may play a critical role in regulating cellular lactate metabolism. Additionally, MRPL3 knockdown caused a decrease in XCT protein expression, and under glucose deprivation conditions, we observed an upregulation of GLUT1 expression, while MRPL3, LDHA, and XCT were consistently downregulated.

We hypothesize that under glucose deprivation stress, cells upregulate GLUT1 to enhance glucose uptake. However, due to substrate deficiency, the overall glycolytic pathway is restricted, resulting in decreased LDHA expression (38). Under normal conditions, sufficient glucose supports the glycolytic-pentose phosphate pathway to produce large amounts of NADPH. This enables the efficient progression of two essential NADPH-dependent processes: the reduction of cystine (imported via XCT) to cysteine for glutathione synthesis, and the regeneration of reduced glutathione (39). Consequently, robust antioxidant function is maintained, promoting tumor cell survival. However, under glucose deprivation, insufficient NADPH supply creates a metabolic vulnerability. Sustained high expression of XCT under such conditions may lead to excessive cystine import without adequate reduction, thereby precipitating disulfidptosis (10). Therefore, this findings suggest that under normal conditions, MRPL3 promotes tumor survival by maintaining XCT expression. In metabolic stress, however, the downregulation of MRPL3 helps cells evade disulfidptosis. In this context, we further observed that MRPL3 knockdown potentiates the endogenous protective response of cells under glucose deprivation, markedly attenuating cytoskeletal damage induced by glucose deprivation and preserving a more intact F-actin architecture. These findings suggest that MRPL3 downregulation-mediated suppression of XCT expression constitutes a key adaptive mechanism that enables cells to resist disulfidptosis and sustain cytoskeletal homeostasis within a glucose-deficient microenvironment. This reveals a context-dependent, dual role for MRPL3 in metabolic adaptation and cell fate determination.

## 5 Conclusion

We have developed and validated a robust prognostic model for LUAD based on seven RGs. This model not only accurately predicts patient survival but also effectively reflects the status of the tumor immune microenvironment and potential therapeutic responses. Our study links the mitochondrial function-related MRPL3 with the immune suppressive features of LUAD, providing new insights into the complex network of tumor metabolism and immune interactions. Furthermore, we demonstrate that MRPL3 may drive tumor progression by modulating glycolytic pathways and disulfidptosis. These findings offer valuable tools for patient stratification and highlight MRPL3 as a potential therapeutic target for LUAD patients.

## Data Availability

The original contributions presented in the study are included in the article/Supplementary Material. Further inquiries can be directed to the corresponding authors.
