# Supplementary material for: MRPL3 is identified as a prognostic biomarker and therapeutic target in lung adenocarcinoma via a lactylation-disulfidptosis gene signature model and experimental validation
Source: Front Immunol. 2026 May 19;17:1772955. doi: 10.3389/fimmu.2026.1772955 (PMC13226520; doi:10.3389/fimmu.2026.1772955)
Supplement: Supplementary file 1 [file Image1.pdf]

Supplementary Figures

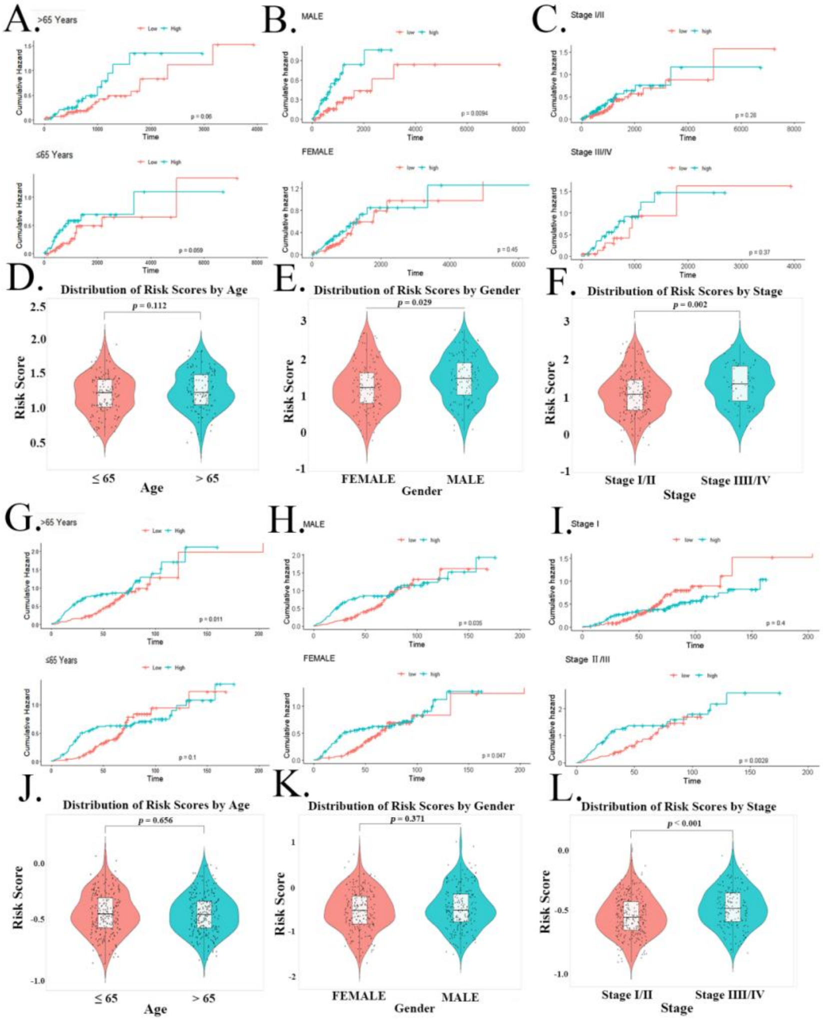

Figure S1: Risk characteristics of clinical variables by subgroup in the TCGA testing set and GSE68465 cohort. Cumulative risk over time in high- and low-risk groups stratified by clinical variables (age, gender, stage) for the (A-C) TCGA training set and (G-I) GSE68465 cohort. Comparison of risk scores across the same clinical subgroups for the (D-F) TCGA training set and (J-L) GSE68465 cohort.

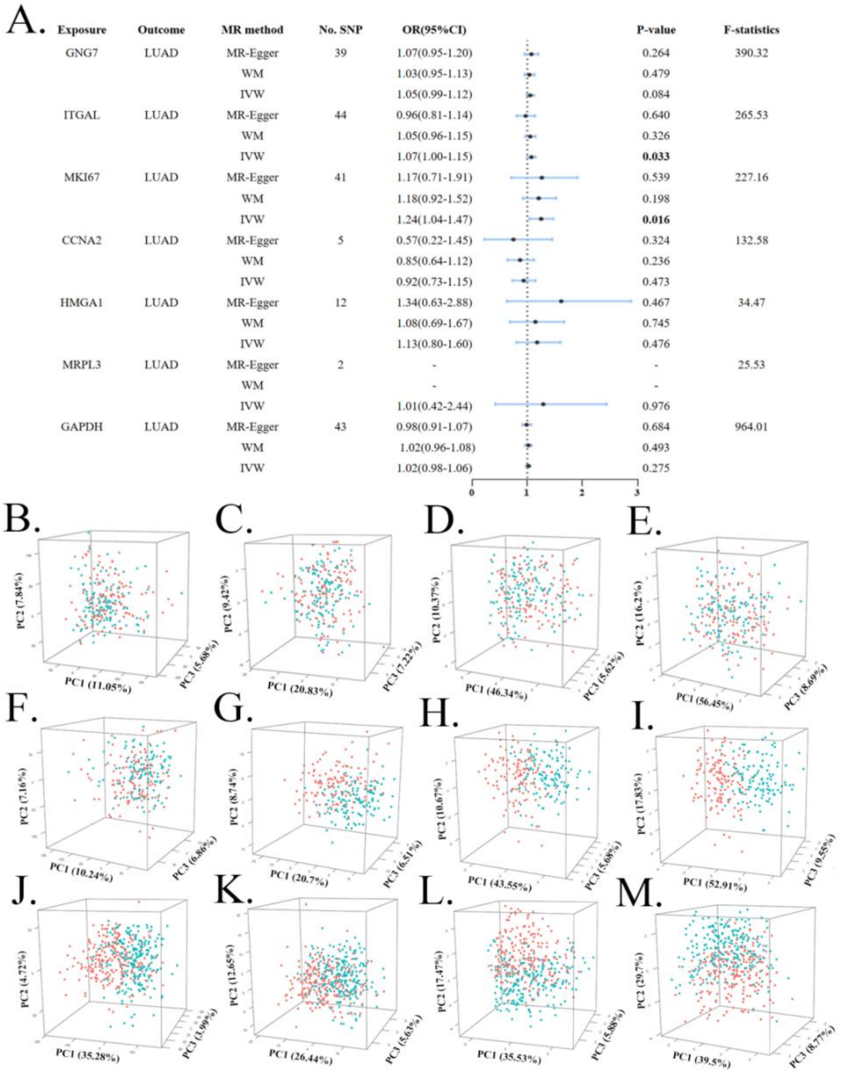

Figure S2: Causal effects of seven RGs on LUAD and PCA of different gene sets. (A) MR analysis reveals the causal effects of the seven RGs on LUAD risk. (B-E) PCA in the TCGA training cohort using four gene sets: (B) all genes, (C) CGs, (D) PGs, and (E) the seven RGs. (F-I) PCA validation in the TCGA testing cohort using the same four gene sets: (F) all genes, (G) CGs, (H) PGs, and (I) the seven RGs. (J-M) PCA validation in the GSE68465 cohort using the same four gene sets: (J) all genes, (K) CGs, (L) PGs, and (M) the seven RGs.

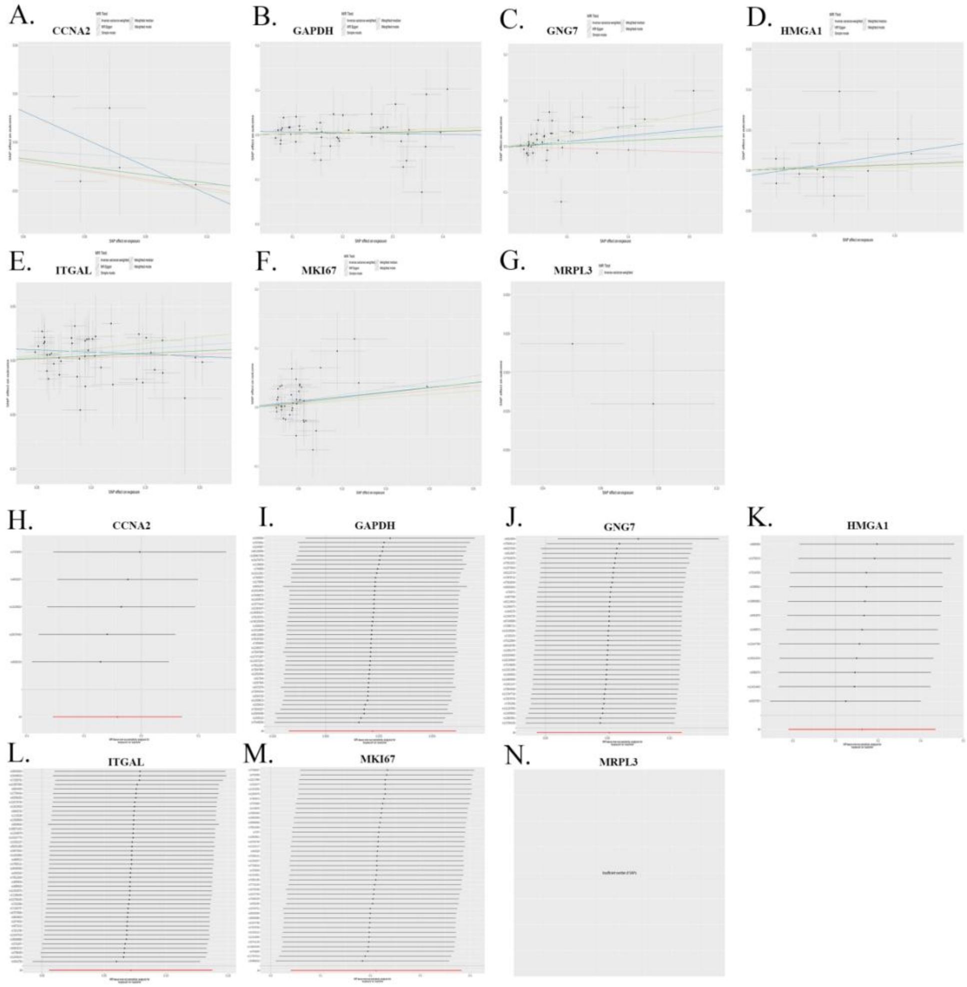

Figure S3: Scatter plots and leave-one-out sensitivity analysis results for the MR.

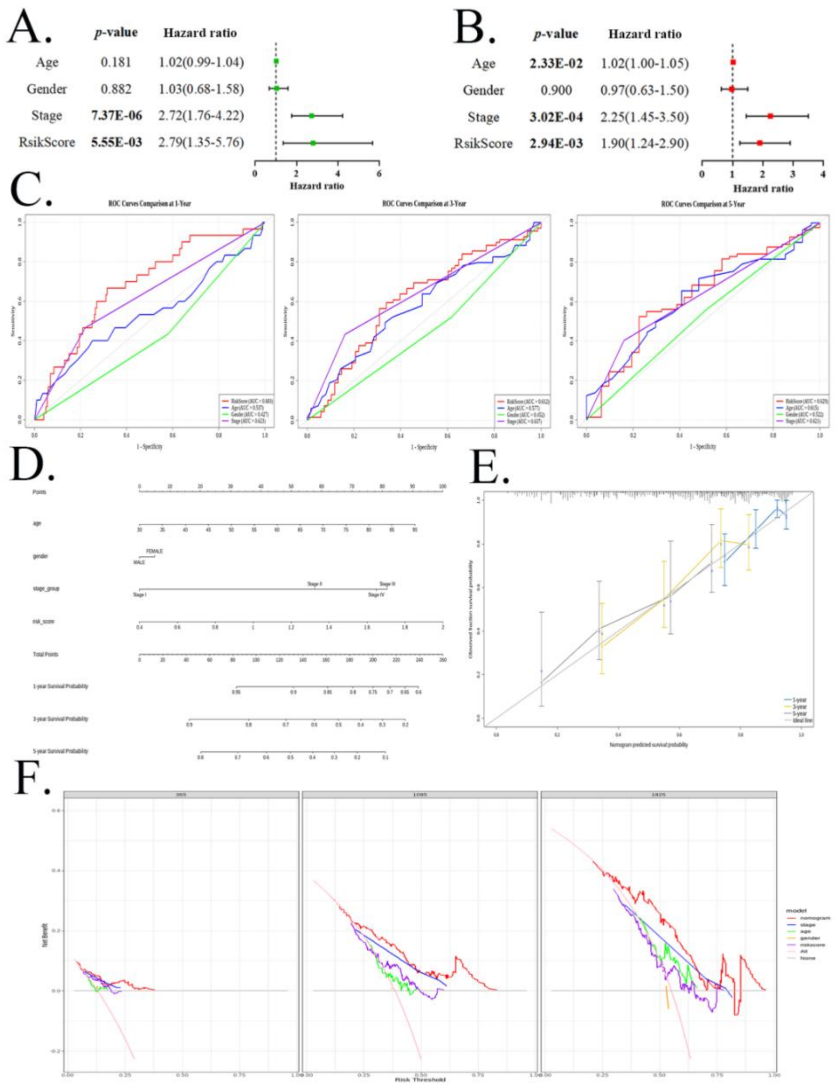

Figure S4: Prognostic model validation and nomogram construction in LUAD testing set. (A) univariate and (B) multivariate Cox regression analyses evaluating the risk score as an independent prognostic factor. (D) ROC curves comparing the predictive accuracy of the risk score against clinical variables (age, gender, stage) for 1-, 3-, and 5-year survival. (E) Nomogram constructed based on the TCGA testing set. (F) Nomogram calibration plots for 1, 3, and 5 years. (G) DCA evaluating the clinical net benefit of the nomogram against individual clinical variables across different time points.

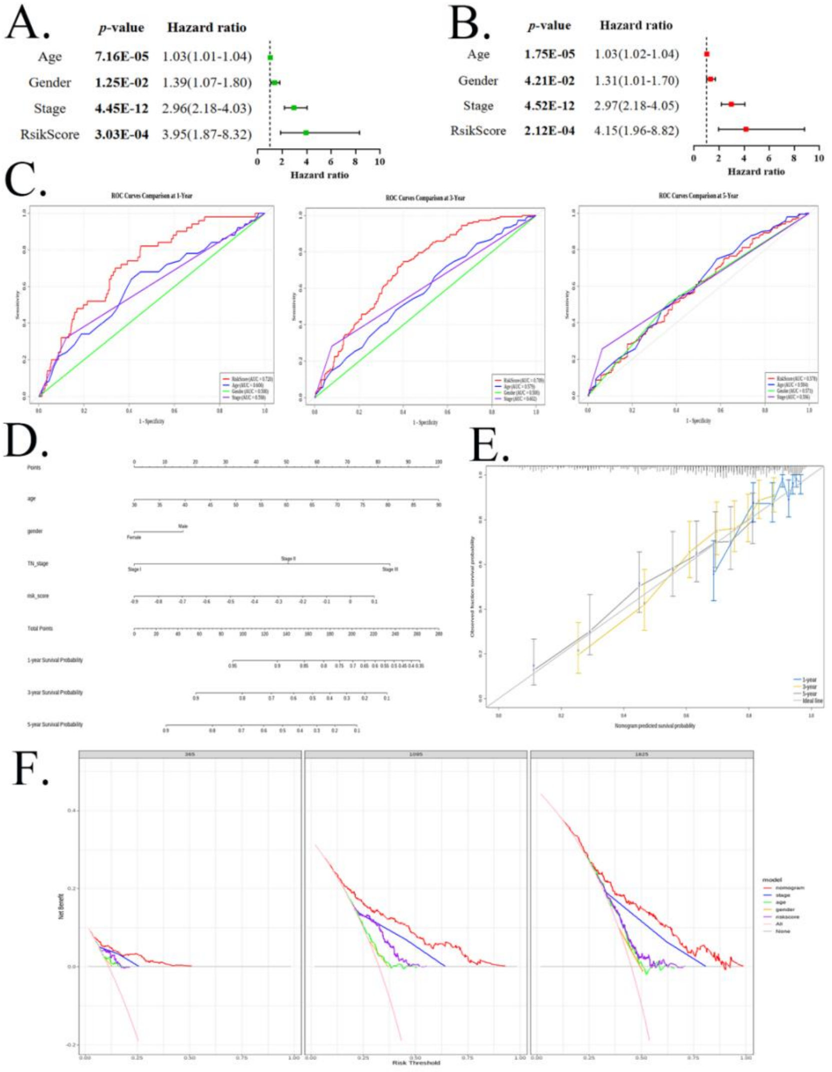

Figure S5: Prognostic model validation and nomogram construction in the GSE68465 cohort. (A) Univariate and (B) multivariate Cox regression analyses assessing the risk score as an independent prognostic factor. (D) ROC curves comparing the predictive accuracy of the risk score with clinical variables (age, gender, stage) for 1-, 3-, and 5-year survival. (E) Nomogram based on the GSE68465 cohort. (F) Calibration plots for 1-, 3-, and 5-year survival. (G) DCA evaluating the clinical benefit of the nomogram compared to clinical variables at different time points.

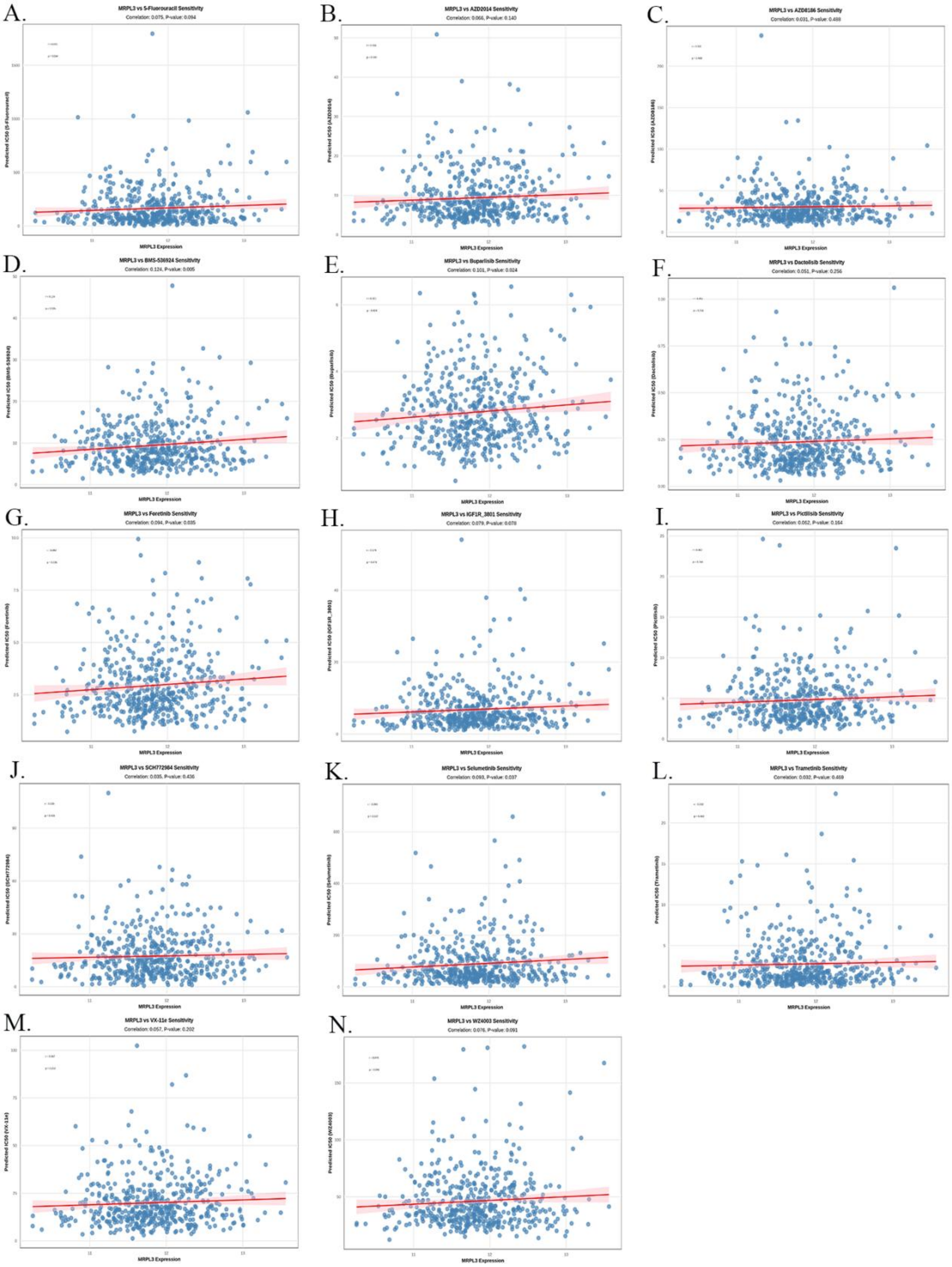

Figure S6: The expression of MRPL3 is positively correlated with the IC50 of drugs showing differential sensitivity between the high-risk and low-risk groups.
