# Supplementary material for: MRPL3 is identified as a prognostic biomarker and therapeutic target in lung adenocarcinoma via a lactylation-disulfidptosis gene signature model and experimental validation
Source: Front Immunol. 2026 May 19;17:1772955. doi: 10.3389/fimmu.2026.1772955 (PMC13226520; doi:10.3389/fimmu.2026.1772955)
Supplement: Supplementary file 3 [file Table2.docx]

Supplementary Table S2. Sensitivity analysis of eQTL

| Exposures | Outcomes | Heterogeneity test | | | | |  | Pleiotropy test | |
| --- | --- | --- | --- | --- | --- | --- | --- | --- | --- |
|  |  | IVW^[[1]](#footnote-0)^ | |  | MR-Egger | |  | MR-Egger intercept | |
|  |  | Q | pval |  | Q | pval |  | Intercept | pval |
| CCNA2 | LUAD | 3.326 | 0.505 |  | 2.252 | 0.522 |  | 0.035 | 0.376 |
| GAPDH | LUAD | 42.829 | 0.435 |  | 41.674 | 0.441 |  | 0.007 | 0.293 |
| GNG7 | LUAD | 29.124 | 0.849 |  | 29.045 | 0.822 |  | -0.002 | 0.780 |
| HMGA1 | LUAD | 7.368 | 0.769 |  | 7.130 | 0.713 |  | -0.009 | 0.636 |
| ITGAL | LUAD | 21.884 | 0.997 |  | 19.862 | 0.999 |  | 0.012 | 0.162 |
| MKI67 | LUAD | 38.612 | 0.533 |  | 38.556 | 00.490 |  | 0.002 | 0.814 |
| MRPL3 | LUAD | 0.474 | 0.491 |  | - | - |  | - | - |

1. IVW, inverse variance weighted [↑](#footnote-ref-0)
