# Supplementary material for: MRPL3 is identified as a prognostic biomarker and therapeutic target in lung adenocarcinoma via a lactylation-disulfidptosis gene signature model and experimental validation
Source: Front Immunol. 2026 May 19;17:1772955. doi: 10.3389/fimmu.2026.1772955 (PMC13226520; doi:10.3389/fimmu.2026.1772955)
Supplement: Supplementary file 4 [file Table3.docx]

| **Clinical_feature** | **Effect_size** | **P_value** | **FDR** |
| --- | --- | --- | --- |
| Age at Diagnosis | -0.0177 | 0.7051 | 0.8227 |
| Pathologic Stage (AJCC) | 0.0408 | <0.001 | 0.0029 |
| Pathologic T Stage (AJCC) | 0.053 | <0.001 | <0.001 |
| Pathologic M Stage (AJCC) | 0.0266 | 0.0029 | 0.0103 |
| Pathologic N Stage (AJCC) | 0.023 | 0.0054 | 0.0151 |
| Vital Status (Dead） | 0.0127 | 0.0124 | 0.029 |
| Gender (male) | 0.0107 | 0.0216 | 0.0433 |

Table S3. Correlation between MRPL3 expression levels and clinicopathological characteristics of LUAD (TCGA)
